# Association of Ezrin expression with the progression and prognosis of gastrointestinal cancer: a meta-analysis

**DOI:** 10.18632/oncotarget.21473

**Published:** 2017-10-04

**Authors:** Feng Liang, Yangxin Wang, Ligen Shi, Jianmin Zhang

**Affiliations:** ^1^ Department of Neurosurgery, Second Affiliated Hospital, School of Medicine, ZheJiang University, HangZhou, ZheJiang 310009, China; ^2^ Department of Orthopaedic Surgery, Second Affiliated Hospital, School of Medicine, ZheJiang University, HangZhou, ZheJiang 310009, China

**Keywords:** ezrin, gastrointestinal, cancer, prognosis

## Abstract

**Background:**

Ezrin, a cytoskeletal protein, is involved in cell adhesion. Several studies have been performed to explore the association between Ezrin and gastrointestinal cancers, but the results are inconclusive. This meta-analysis aims to assess the prognostic value of Ezrin.

**Materials and Methods/Findings:**

PubMed and EMBASE were searched. Pooled hazard ratio (HR), odds ratio (OR) and 95% confidence intervals (CI) were utilized to evaluate the association between Ezrin expression and various clinical parameters. 2701 patients from 19 studies were included in the meta-analysis. For gastric cancer, Ezrin expression was associated with tumor grade (OR 2.32, 95% CI 1.53–3.52), TNM stage (OR 4.69, 95% CI 1.38–15.89), lymph node involvement (OR 3.96, 95% CI 1.47–10.70) and overall survival (HR 1.88, 95% CI 1.33–2.66). In colorectal cancer, Ezrin expression was associated with tumor grade (OR 3.94, 95% CI 2.10–3.78), TNM stage (OR 5.66, 95% CI 1.41–22.67), lymph node metastasis (OR 9.52, 95% CI 3.93–23.02), distant metastasis (OR 3.06, 95% CI 1.77–5.31), disease free survival (HR 2.48, 95% CI 1.44–4.28). For esophageal cancer, Ezrin expression was associated with lymph node metastasis (OR 2.07, 95% CI 1.00–4.28) and overall survival (HR 1.49, 95% CI 1.17–1.89).

**Conclusions:**

Ezrin expression is significantly associated with tumor grade, TNM stage, and lymph node metastasis in gastric and colorectal cancers. For gastric cancers, Ezrin is useful in predicting distant metastasis. Survival data showed that high Ezrin expression is associated with poor prognosis in gastric, colorectal and esophageal cancers. Our findings suggest that Ezrin might be a potential biomarker in several gastrointestinal cancers.

## INTRODUCTION

Gastrointestinal cancers include colorectal, gastric and esophageal malignancies. They are very common worldwide, with high mortality rates [1, 2]. Early diagnosis and treatment could improve the outlook of these patients. However, there exists no ideal biomarker for diagnosing and predicting the prognosis of gastrointestinal cancers. Thus, it is important to identify molecular biomarkers to identify individuals at high risk of developing, and also for predicting prognosis in patients diagnosed with, gastrointestinal cancers.

Ezrin, a cytoskeletal protein, is a member of the ezrin/radixin/moesin (ERM) family. It is involved in a number of signaling pathways that is crucial to cancer progression [3]. For example, Ezrin provides a regulated linkage between the actin in cytoskeleton and the cell membrane [3, 4]. The attachment of F-actin to the cell membrane is essential for many cellular processes, including the determination of cell shape and surface structures, cell adhesion, motility, migration, cytokinesis, phagocytosis, membrane transport and signal transduction [4–7]. In aggregate, these functions suggest that Ezrin plays a pivotal role in the development and progression of cancer. Previous studies have reported that Ezrin overexpression was associated with poor prognosis in many malignancies such as ovary cancer [8], salivary gland cancer [9], breast cancer [10], colorectal cancer [11], hepatocellular cancer [12] and lung cancer [13]. However, the significance of Ezrin in gastrointestinal cancers remains unclear. In the present study, we performed a meta-analysis to explore the possible role of Ezrin expression in the progression and prognosis of gastric, colorectal and esophageal cancers.

## RESULTS

### Characteristics of studies

**Figure 1:**
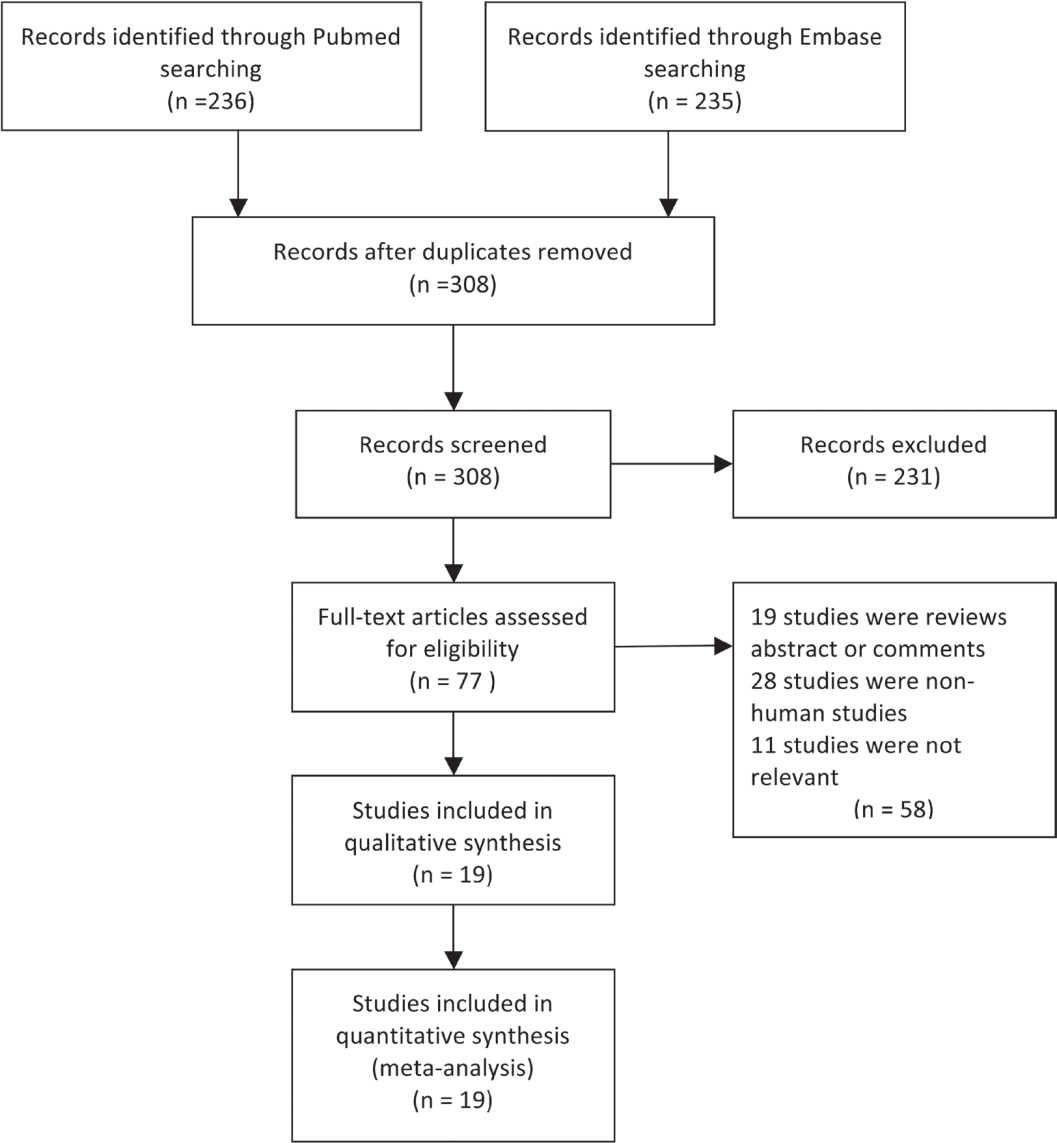
Flow chart of the study selection process

A total of 471 studies were retrieved using the strategy described above. 452 studies were excluded after careful screening. The detail of the screening process was shown in Figure 1. In the end, 19 studies [14–32] with 2701 patients were included in the meta-analysis (Figure 1).

In total, 7 studies each yielded data on Ezrin expression in gastric and colorectal cancers, while 5 studies provided information on Ezrin expression and esophageal cancer. The characteristics of the included studies are summarized in Table 1. The total number of patients included for the meta-analysis was 2701. The number of patients with ‘high’ Ezrin was 1551 (57.4%).

**Table 1:** Characteristics of studies for association between Ezrin and different gastrointestinal cancer

Source	Year	Tumor type (histotype)	No of patients	Sample	Technology	Cut-off level of high Ezrin expressin	No of patients with high Ezrin	Study quality (points)
Shi [14]	2006	Gastric (Adenocarcinoma)	90	TMA	IHC	IRS ≥ 7 (0–7)	48	6/9
Wang [15]	2007	Colorectal (Adenocarcinoma)	50	WTS	IHC	IRS ≥ 2 (0–2)	37	6/9
Ge [16]	2007	Colorectal (Adenocarcinoma)	132	WTS	IHC	> 10%^*^	105	7/9
Chai [17]	2007	Esophageal (ESCC)	71	TMA	IHC	IRS ≥ 5 (0–12)	51	6/9
Bal [18]	2007	Gastric (Adenocarcinoma)	75	WTS	IHC	IRS ≥ 1 (0–12)	52	7/9
Zhang [19]	2008	Gastric (^#^Mixed)	80	WTS	IHC	> 50%^*^	49	6/9
Elzagheid [20]	2008	Colorectal (Adenocarcinoma)	74	WTS	IHC	IRS ≥ 2 (0–3)	62	8/9
Wang [21]	2009	Colorectal (Adenocarcinoma)	80	WTS	IHC	IRS ≥ 2 (0–2)	60	6/9
Zhai [22]	2009	Esophageal (ESCC)	76	TMA	IHC	> 50%^*^	34	6/9
Gao [23]	2009	Esophageal (ESCC)	193	TMA	IHC	> 50%^*^	90	8/9
Patara [24]	2011	Colorectal (Adenocarcinoma)	250	TMA	IHC	IRS ≥ 2 (0–3)	21	8/9
Xie [25]	2011	Esophageal (ESCC)	307	TMA	IHC	IRS ≥ 5 (0–12)	240	8/9
Li [26]	2011	Gastric (Adenocarcinoma)	436	TMA	IHC	IRS ≥ 4 (0–9)	236	8/9
Lam [27]	2011	Gastric (Adenocarcinoma)	150	TMA	IHC	IRS ≥ 4 (0–9)	117	8/9
Korkeila [28]	2011	Colorectal (Adenocarcinoma)	76	WTS	IHC	IRS ≥ 2 (0–3)	33	8/9
Jorgren [29]	2012	Colorectal (NA)	104	TMA	IHC	IRS ≥ 3 (0–3)	22	7/9
Jin [30]	2012	Gastric (Adenocarcinoma)	277	TMA	IHC	IRS ≥ 2 (0–3)	168	7/9
Tobo [31]	2013	Gastric (Adenocarcinoma)	104	WTS	IHC	> 30%^*^	95	6/9
Zhai [32]	2014	Esophageal (ESCC)	76	TMA	IHC	> 50%^*^	31	6/9

^*^: Percentage with strong expression. Abbreviations: ESCC, esophageal squamous cell carcinoma; NA, histotype not available; ^#^Mixed: 72 patients with adenocarcinoma, 5 patients with adenosquamous carcinoma and 3 patients with undifferentiated carcinoma were recruited in this study; TMA, tissue microarray; WTS, whole tissue section; IHC, immunohistochemistry; IRS, immunoreactive score.

### Ezrin expression and gastric cancer

There were 7 studies with data on Ezrin expression and gastric cancer. Respectively, 6/4/6/3 studies had data regarding Ezrin expression and tumor grade/TNM stage/lymph node involvement/distant metastasis in gastric cancer.

**Figure 2:**
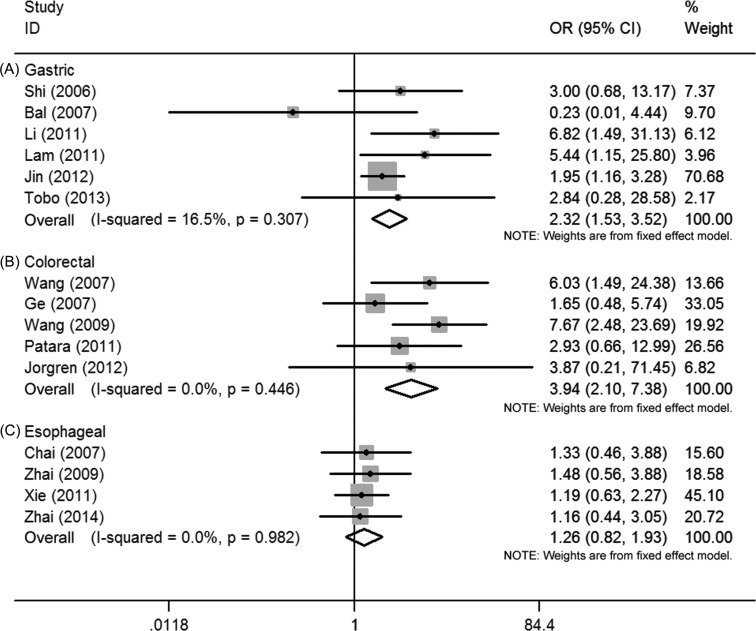
Forest plot of the association between Ezrin and tumor grade in (**A**) gastric cancer, (**B**) colorectal cancer and (**C**) esophageal cancer.

**Figure 3:**
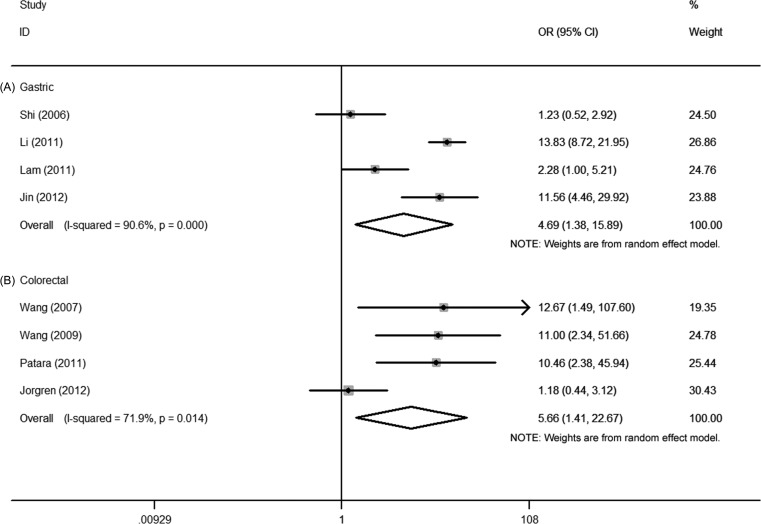
Forest plot of the association between Ezrin and TNM stage in (**A**) gastric cancer and (**B**) colorectal cancer.

**Figure 4:**
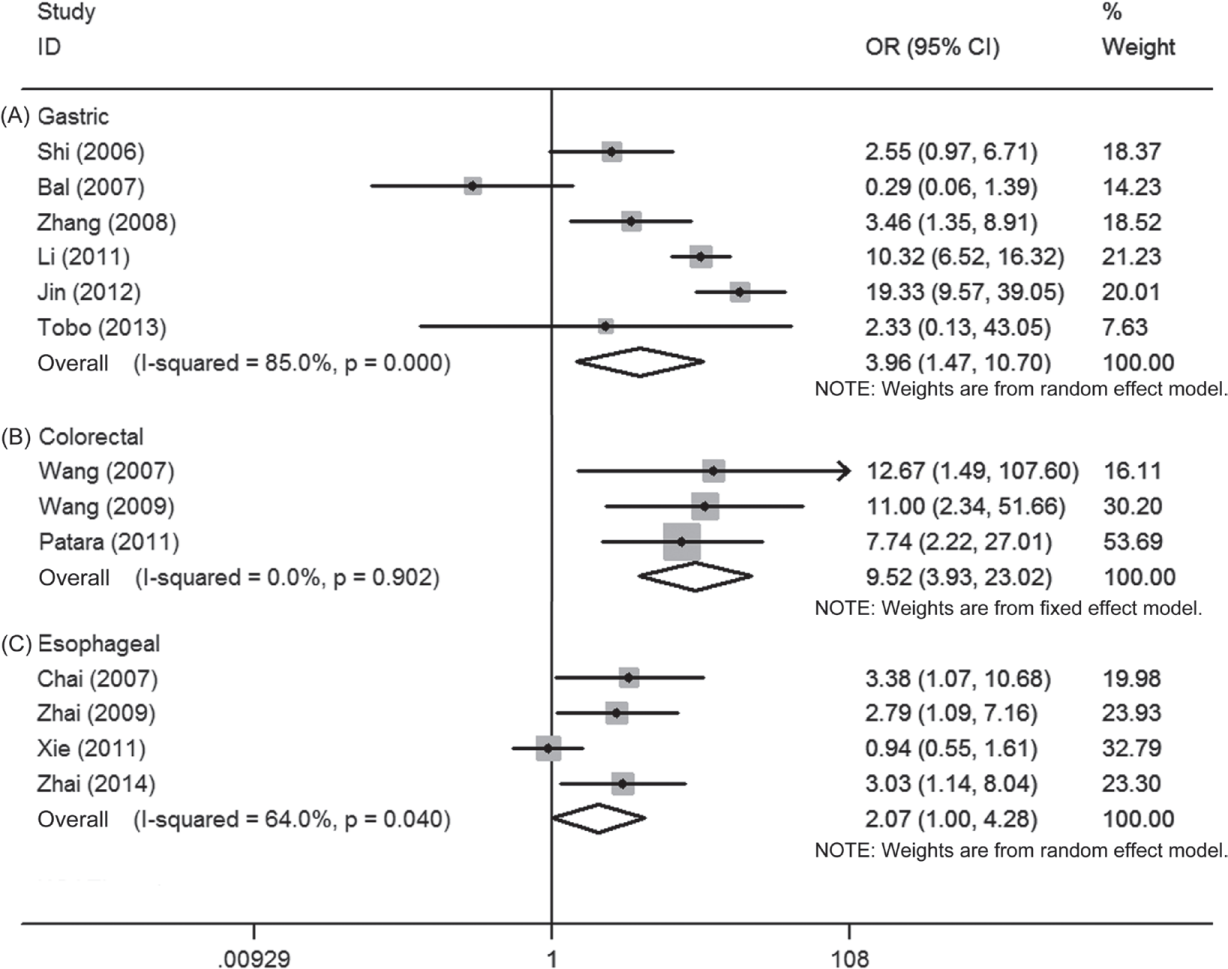
Forest plot of the association between Ezrin and lymph node metastasis in (**A**) gastric cancer, (**B**) colorectal cancer and (**C**) esophageal cancer.

**Figure 5:**
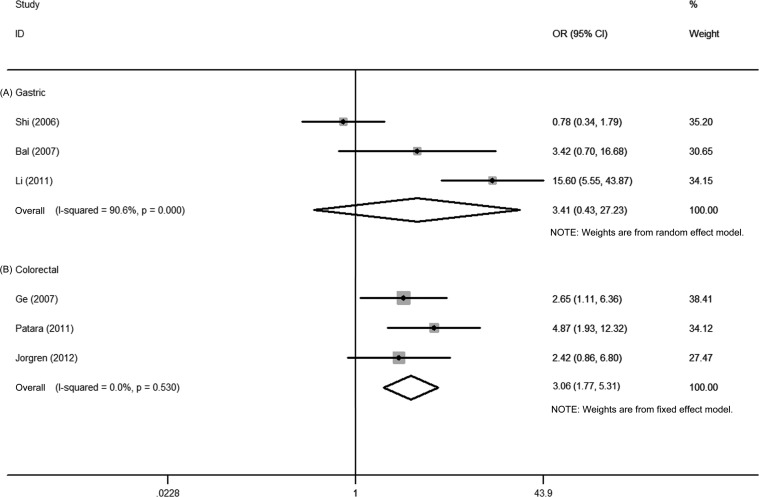
Forest plot of the association between Ezrin and distant metastasis in (**A**) gastric cancer and (**B**) colorectal cancer.

First, the pooled data of 6 studies showed that high Ezrin expression was significantly associated with tumor grade in gastric cancer (OR = 2.32, 95% CI = 1.53–3.52, *P* = 0.000). No significant heterogeneity among these 6 studies was observed (*I^2^* = 16.5%, *P* = 0.307) (Figure 2A). Second, the pooled data from 4 studies showed that high Ezrin expression was significantly associated with the TNM stage of gastric cancer (OR = 4.69, 95% CI = 1.38–15.89, *P* = 0.013), but the heterogeneity among these 4 studies was significant (*I^2^* = 90.6%, *P* = 0.000) (Figure 3A). Third, the pooled data from 6 studies showed that high Ezrin expression was significantly associated with lymph node metastasis in gastric cancer (OR = 3.96, 95% CI = 1.47–10.70, *P* = 0.007), with significant heterogeneity among studies (*I^2^* = 85.0%, *P* = 0.000) (Figure 4A). In addition, the pooled data of 3 studies showed that high Ezrin expression was not associated with distant metastasis in gastric cancer (OR = 3.41, 95% CI = 0.43–27.23, *P* = 0.247). There, significant heterogeneity among the 3 studies was observed (*I^2^* = 90.6%, *P* = 0.000) (Figure 5A).

Sensitivity analysis was performed to evaluate the stability of pooled results by sequentially removing individual studies. No single study qualitatively changed the pooled OR. Particularly, 6 out of 7 studies recruited patients with gastric adenocarcinoma. The rest one (Zhang [19]) included multiple histological types (Table 1). Detailed information about Ezrin expression in each histotype was not provided by this article, but sensitivity analysis by removing this article did not significantly influenced the pooled results (Supplementary Materials).

### Ezrin expression and colorectal cancer

There were 7 studies concerning Ezrin expression and colorectal cancer. Respectively, 5/4/3/3 studies provided data on Ezrin expression and tumor grade/TNM stage/lymph node involvement/distant metastasis in colorectal cancer.

First, the pooled data of 5 studies showed that high Ezrin expression was significantly associated with tumor grade in colorectal cancer (OR = 3.94, 95% CI = 2.10–7.38, *P* = 0.000), where no significant heterogeneity among studies was observed (*I^2^* = 0.0%, *P* = 0.446) (Figure 2B). Second, the pooled data of 4 studies showed significant association between high Ezrin expression and the TNM stage of colorectal cancer (OR = 5.66, 95% CI = 1.41–22.67, *P* = 0.014). But heterogeneity among these studies was significant (*I^2^* = 71.9%, *P* = 0.014) (Figure 3B). Third, the combined data of 3 studies showed that high Ezrin expression was significantly associated with lymph node metastasis in colorectal cancer (OR = 9.52, 95% CI = 3.93–23.02, *P* = 0.000), with no significant heterogeneity among studies observed (*I^2^* = 0.0%, *P* = 0.902) (Figure 4B). Also, the pooled data of 3 studies showed that high Ezrin expression was significantly associated with distant metastasis in colorectal cancer (OR = 3.06, 95% CI = 1.77–5.31, *P* = 0.000), where no significant heterogeneity among studies was noted (*I^2^* = 0.0%, *P* = 0.530) (Figure 5B).

Sensitivity analysis suggested that the result was reliable. Particularly, 6 out of 7 studies recruited patients with colorectal adenocarcinoma. The rest one (Jorgren [29]) did not provide detailed data on histological type, but sensitivity analysis by removing this article did not significantly influenced the pooled results (Supplementary Materials).

### Ezrin expression and esophageal cancer

In total, 5 studies provided data on Ezrin expression and esophageal cancer. Respectively, 4/1/4/1 studies were examined for any association between Ezrin expression and tumor grade/TNM stage/lymph node involvement/distant metastasis in esophageal cancer.

The pooled data of 4 studies showed that high Ezrin expression was not significantly associated with tumor grade in esophageal cancer (OR = 1.26, 95% CI = 0.82–1.93, *P* = 0.290), where no significant heterogeneity among studies was observed (I2 = 0.0%, *P* = 0.982) (Figure 2C). The pooled data of 4 studies showed significant association between high Ezrin expression and lymph node metastasis in esophageal cancer (OR = 2.07, 95% CI = 1.00–4.28, *P* = 0.050). The heterogeneity among these studies was significant (*I*^2^ = 64.0%, *P* = 0.040) (Figure 4C). Only one study reported on Ezrin expression and TNM stage/distant metastasis in esophageal cancer [25]. It showed that high Ezrin expression was not significantly associated with TNM stage (OR = 1.14, 95% CI = 0.66–1.97, *P* = 0.631) or distant metastasis (OR = 1.32, 95% CI = 0.37–4.74, *P* = 0.669) in esophageal cancer.

Sensitivity analysis showed that no single study significantly influenced the pooled results (Supplementary Materials).

### Ezrin expression and survival

In total, 6 studies were employed to examine possible association between Ezrin expression and survival.

The pooled data of two studies showed that high Ezrin expression was significantly associated with overall survival (OS) for gastric cancer (HR = 1.88, 95% CI = 1.33–2.66, *P* = 0.000), and no significant heterogeneity was observed (*I*^2^ = 0.0%, *P* = 0.446) (Table 2).

**Table 2:** The association between Ezrin and survival in different gastrointestinal cancer

Cancer	No of study	Pooled HR	95% CI	*P* Value	Heterogeneity		
					Statistic model	*I*^2^ (%)	*P* Value
**Esophageal**							
OS	2	1.49	1.17–1.89	0.001	Fixed	0.0	0.914
**Gastric**							
OS	2	1.88	1.33–2.66	0.000	Fixed	0.0	0.446
**Colorectal**							
DFS	2	2.48	1.44–4.28	0.001	Fixed	0.0	0.759

Two studies examined Ezrin expression and disease-free survival (DFS) in colorectal cancer. The pooled data showed significant association (HR = 2.48, 95% CI = 1.44–4.28, *P* = 0.001), with no significant heterogeneity observed (*I^2^* = 0.0%, *P* = 0.759) (Table 2).

Significant association was also observed between high Ezrin expression and OS in esophageal cancer (HR = 1.49, 95% CI = 1.17–1.89, *P* = 0.001), with no significant heterogeneity noted (*I^2^* = 0.0%, *P* = 0.914) (Table 2).

### Publication bias

In this present meta-analysis, both Begg's and Egger's *P*-value tests were used to assess publication bias. No evidence of significant publication bias was found (Supplementary Materials).

## DISCUSSION

The basic role where Ezrin interacts with membrane protein and cytoskeleton actin was mentioned before. Several studies have been performed to evaluate the role played by Ezrin in gastrointestinal cancers. Heiska *et al*. [33] demonstrated that Ezrin is one of the downstream targets of Src and Src, which in turn leads to deregulation of cell-cell adhesion and actin cytoskeleton in colon cancer [34]. Lam *et al*. [28] reported that Ezrin upregulation leads to aberrant Ras activation in gastric cancer. Pujuguet *et al*. [7] showed that Ezrin regulates the transmission of Ecadherin to cell membrane, and is involved in the dergulation of E-cadherin. The latter is believed to promote tumor invasion [35], but the underlying molecular mechanism remains unclear. Ezrin also takes part in several signaling pathways, such as Wnt/β-catenin, PI3K/Akt and CD44, all of which are associated with cancer progression [3]. However, the role played by Ezrin in these pathways is not clear. Therefore, the specific role of Ezrin in different cancers remains to be elucidated.

There is no doubt that effective prognostic factors are very important for clinicians to tailor treatments for patients. The prognostic value of Ezrin has been reported in osteosarcoma [36, 37]. Considering the importance of Ezrin in tumor growth, we focused on the relationship between Ezrin and different gastrointestinal cancers. In this meta-analysis, we included 19 studies with a total of 2701 patients.

Seven eligible studies on gastric cancer met our inclusion criteria. Our results showed significant association between high Ezrin expression and multiple clinical parameters including tumor grade, TNM stage and lymph node metastasis in gastric cancer. We also found that high Ezrin expression was significantly associated with OS in gastric cancer. It means that Ezrin could be a potential biomarker for predicting prognosis in gastric cancer.

Meta-analysis of 7 studies on colorectal cancer showed significant association between high Ezrin expression and tumor grade, TNM stage, lymph node involvement and distant metastasis. In addition, Ezrin expression was found to be significantly associated with DFS in colorectal cancer. These results showed that Ezrin might serve as a prognostic biomarker for colorectal cancer.

Our results showed that high Ezrin expression was not significantly associated with tumor grade in esophageal cancer. But pooled data on esophageal cancer showed significant association between high Ezrin expression and lymph node metastasis. Besides, Ezrin expression was also found to be significantly associated with OS in esophageal cancer. Thus, Ezrin may still be useful in predicting survival of these patients.

Heterogeneity was significant in the meta-analysis of lymph node metastasis, distant metastasis and TNM stage. In this study, histological subtypes, publication year and cut-off standard of Ezrin expression could account for heterogeneity. Therefore, we conducted sensitivity analysis and found that the overall trend remains unchanged when studies was removed one by one. This indicates the results are reliable.

Several limitations exist in the current meta-analysis. First, only studies in Chinese and English were included. The results might be different if reports in other languages are included. Secondly, the number of studies included in the meta-analysis could be higher. Only two studies were included for each cancer in OS/DFS analysis. An insufficient number of patients precluded subgroup analyses based on potential confounding factors within each cancer. More studies need to be included to make our results more robust. Thirdly, the phosphorylation of Ezrin is important in cancer development, but the included studies only evaluated a simple expression of Ezrin. Future studies should include data on the phosphorylation of Ezrin. Finally, the cut-off values for Ezrin expression were different between studies. It is an important factor influencing the results and could have contributed to heterogeneity. We conducted subgroup analysis based on different cut-off values but were unsuccessful due to the limited number of studies.

## MATERIALS AND METHODS

### Publication search

This meta-analysis was conducted according to the Preferred Reporting Items for Systematic Reviews and Meta-Analyses (Supplmentary Table 2 PRISMA) guidelines (Supplementary Materials) [38]. We searched PubMed and EMBASE to identify eligible studies published through 31 Dec 2016. The search was undertaken using the following terms: “ezrin” AND (“gastrointestinal” OR “colorectal” OR “gastric” OR “esophageal”). To insure all relevant studies have been included in this review, in addition to the electronic database search, reference lists from randomized controlled trials and systematic reviews were manually screened.

### Inclusion and exclusion criteria

Inclusion criteria for this study are: (1) patients had confirmed pathological diagnosis of gastrointestinal cancer; (2) Ezrin expression was detected by immunohistochemical methods; (3) the association between Ezrin expression and tumor grade, differentiation, metastasis, overall survival (OS) or disease-free survival (DFS) were evaluated; (4) sufficient data were available for calculation of Hazard ratio (HR), Odds ratio (OR) and 95% confidence interval (95% CI). Duplication of data was avoided by carefully keeping track of the names of all authors and institutions involved in each article. Conference abstracts, case reports, reviews, editorial letters and comments were excluded.

### Data extraction and quality assessment

Two investigators (Feng Liang and Yangxin Wang) independently extracted data from the relevant articles. Disagreements were resolved through discussion.

After strict selection and evaluation, the data were extracted from the included studies as follows: author names, publication years, tumor types, number of patients, Ezrin measurement method, and cut-off used for assessing Ezrin overexpresssion.

The Newcastle–Ottawa Scale [39] was used to access the quality of the included studies by two authors (Feng Liang and Ligen Shi). Studies with NOS scores > 6 were considered to be high quality.

### Statistical analysis

Odds ratios (ORs) with 95% confidence interval (CI) are used to evaluate for association between Ezrin expression and several clinical parameters. Hazard ratios (HRs) with 95% CI are utilized to evaluate the association between Ezrin expression and patient prognosis, including overall survival (OS) and disease free survival (DFS). For those where HRs are not given directly, we read Kaplan-Meier curves by Engauge Digitizer version 4.1 and reconstruct the HRs with 95% CI according to the methods described by Tierney *et al*. [40]. Heterogeneity is assessed using Cochrane's *Q* test and *I^2^* measurement. *P* < 0.05 or *I^2^* > 50% indicates significant heterogeneity. Random-effects model is used if the heterogeneity is significant. Otherwise, a fixed-effect model is used. Sensitivity analysis is conducted to help explain any significant heterogeneity among the studies. Egger's and Begg's tests are used to examine publication bias. All statistical analyses are performed using STATA version 11.0 (STATA Corporation, College Station, TX, USA), and all *P* values are two-sided.

## CONCLUSIONS

This is the first meta-analysis to evaluate the prognostic significance of Ezrin in different gastrointestinal cancers. First, this study indicates that high Ezrin expression is related to tumor grade/TNM stage/lymph node metastasis in patients with gastric/colorectal cancer. Second, Ezrin is capable of predicting distant metastasis in colorectal cancer. Furthermore, high Ezrin expression is associated with poor prognosis according to the pooled data of OS and DFS in gastric/colorectal/esophageal cancers. Therefore, Ezrin may serve as a new biomarker in these cancers to guide clinicians in choosing the most suitable treatment plans. It may also be a potential therapeutic target in the future. However, the limitations mentioned above should be kept in mind. Additional studies with more standardized methodology are needed to confirm our results.

## SUPPLEMENTARY MATERIALS FIGURES AND TABLES




