# Supplementary material for: Association of Ezrin expression with the progression and prognosis of gastrointestinal cancer: a meta-analysis
Source: Oncotarget. 2017 Oct 4;8(54):93186–95. doi: 10.18632/oncotarget.21473 (PMC5696254; doi:10.18632/oncotarget.21473)
Supplement: Supplementary file 1 [file oncotarget-08-93186-s001.pdf]

Association of Ezrin expression with the progression and prognosis of gastrointestinal cancer: a meta-analysis

SUPPLEMENTARY MATERIALS

Esophageal tumor grade

A Publication bias:

```
Begg's Test

  adj. Kendall's Score (P-Q) =         2
          Std. Dev. of Score =      2.94
           Number of Studies =         4
                          z  =      0.68
                   Pr > |z|  =     0.497
                          z  =      0.34 (continuity corrected)
                   Pr > |z|  =     0.734 (continuity corrected)
```

Egger's test

Std_Eff	Coef.	Std. Err.	t	P>|t|	[95% Conf.	Interval]
slope	.0019773	.2586169	0.01	0.995	-1.110761	1.114716
bias	.5340893	.5910855	0.90	0.462	-2.009146	3.077325

B Sensitivity analysis:

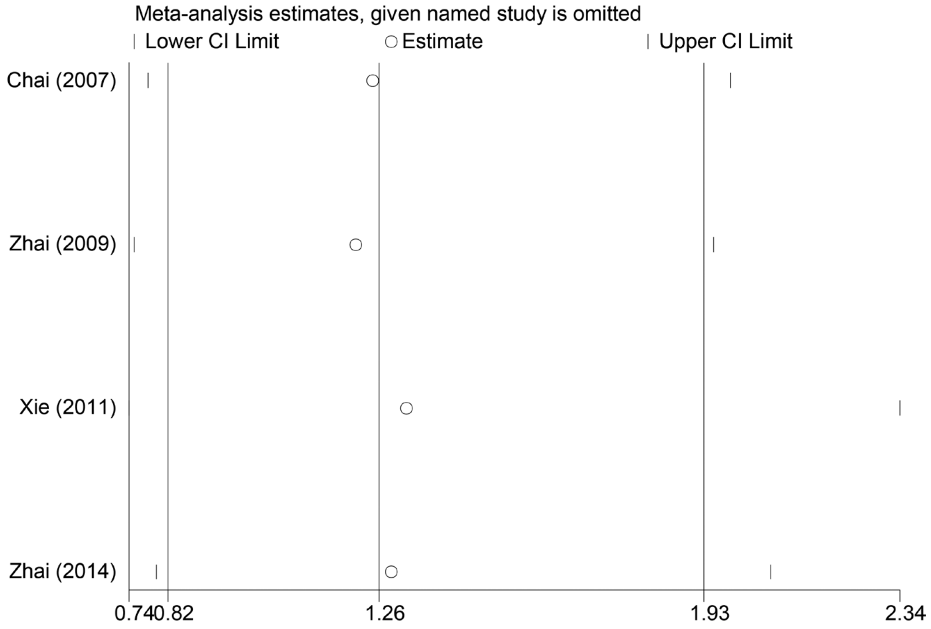

Supplementary Figure 1: (**A**) No evidence of significant publication bias was found in esophageal tumor grade. (**B**) Sensitivity analysis showed that no single study significantly influenced the pooled results of esophageal tumor grade.

Esophageal lymph node metastasis

A Publication bias：

```
Begg's Test

  adj. Kendall's Score (P-Q) =         4
          Std. Dev. of Score =      2.94
           Number of Studies =         4
                          z  =      1.36
                    Pr > |z| =     0.174
                          z  =      1.02 (continuity corrected)
                    Pr > |z| =     0.308 (continuity corrected)
```

Egger's test

Std_Eff	Coef.	Std. Err.	t	P>|t|	[95% Conf.	Interval]
slope	-1.344588	.22603	-5.95	0.027	-2.317116	-.372059
bias	4.713687	.5576793	8.45	0.014	2.314186	7.113187

B Sensitivity analysis：

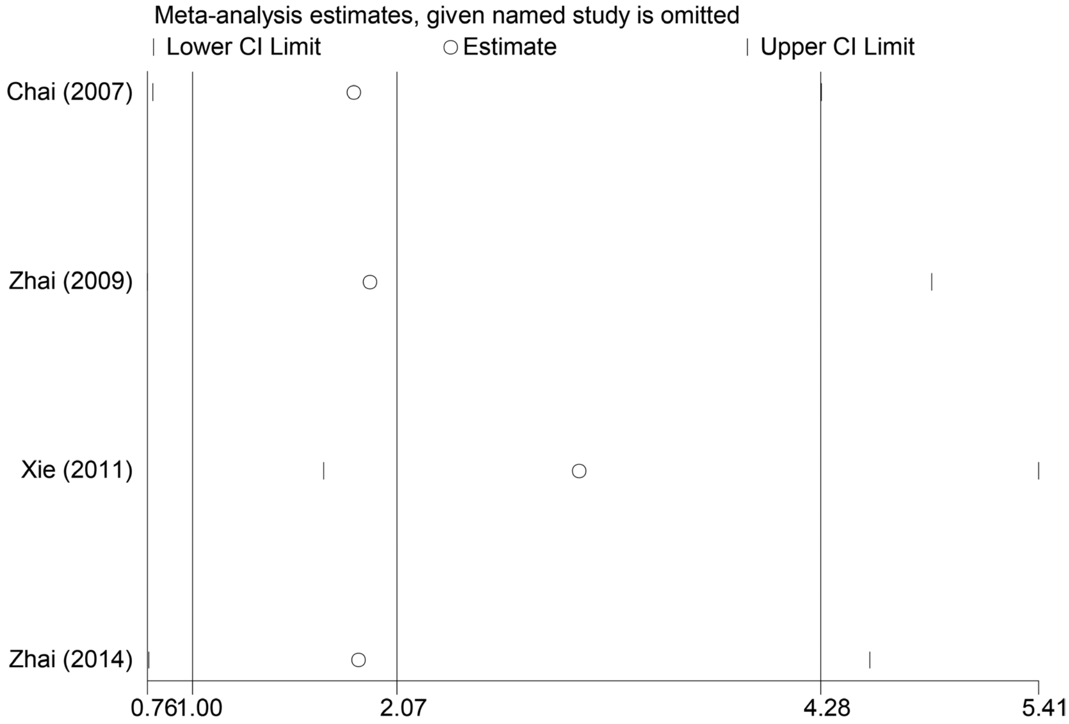

Supplementary Figure 2: (**A**) No evidence of significant publication bias was found in esophageal lymph node metastasis. (**B**) Sensitivity analysis showed that no single study significantly influenced the pooled results of esophageal lymph node metastasis.

Gastric tumor grade

A Publication bias:

```
Begg's Test

  adj. Kendall's Score (P-Q) =        -3
          Std. Dev. of Score =      5.32
           Number of Studies =         6
                          z  =     -0.56
                    Pr > |z| =     0.573
                          z  =      0.38 (continuity corrected)
                    Pr > |z| =     0.707 (continuity corrected)
```

Egger's test

Std_Eff	Coef.	Std. Err.	t	P>|t|	[95% Conf.	Interval]
slope	.6966033	.4727092	1.47	0.215	-.6158479	2.009055
bias	.3543351	.8720404	0.41	0.705	-2.066837	2.775507

B Sensitivity analysis:

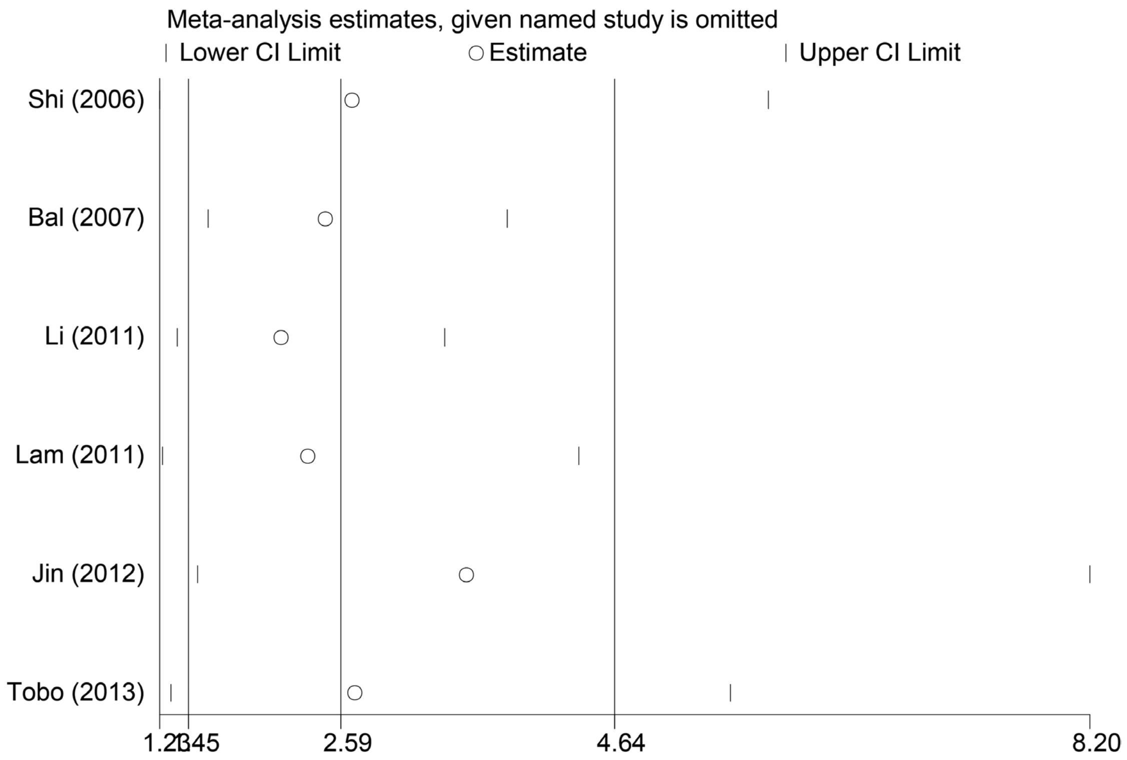

Supplementary Figure 3: (**A**) No evidence of significant publication bias was found in gastric tumor grade. (**B**) Sensitivity analysis showed that no single study significantly influenced the pooled results of gastric tumor grade.

Gastric TNM stage

A Publication bias:

```
Begg's Test

  adj. Kendall's Score (P-Q) =       -2
          Std. Dev. of Score =     2.94
           Number of Studies =        4
                          z  =    -0.68
                 Pr > |z| =    0.497
                          z  =     0.34 (continuity corrected)
                 Pr > |z| =    0.734 (continuity corrected)
```

Egger's test

Std_Eff	Coef.	Std. Err.	t	P>|t|	[95% Conf.	Interval]
slope	4.04586	1.639618	2.47	0.132	-3.008846	11.10057
bias	-6.414704	4.712096	-1.36	0.306	-26.68922	13.85981

B Sensitivity analysis:

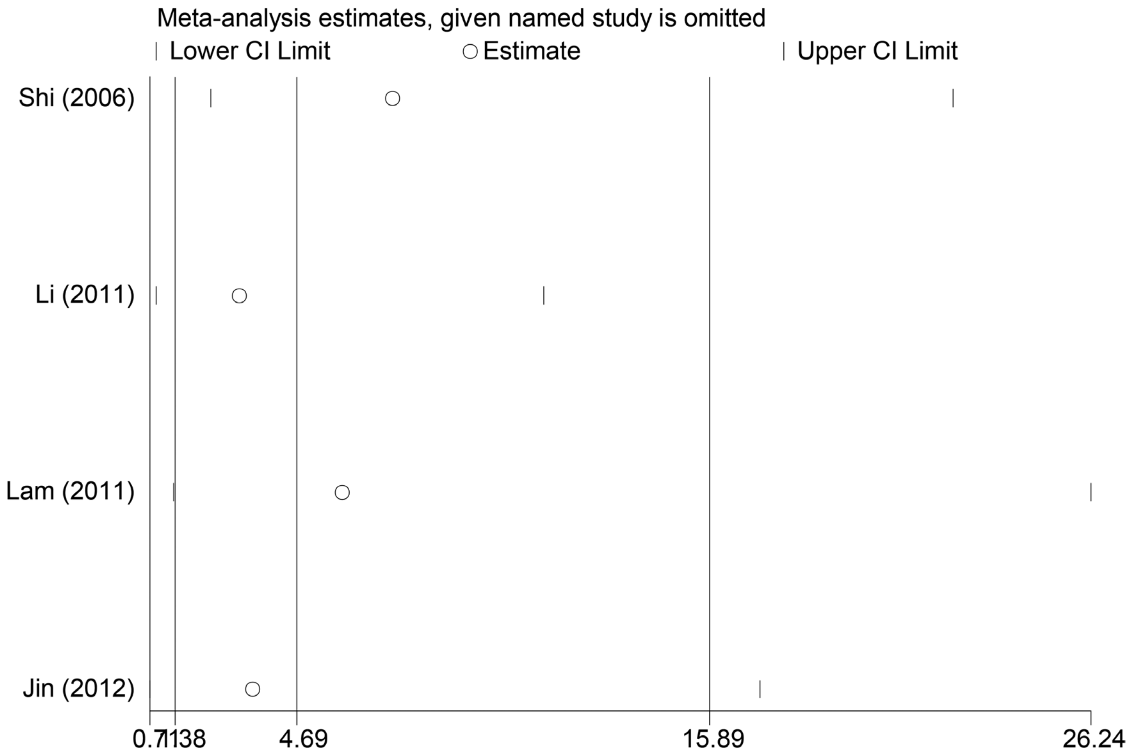

Supplementary Figure 4: (**A**) No evidence of significant publication bias was found in gastric TNM stage. (**B**) Sensitivity analysis showed that no single study significantly influenced the pooled results of gastric TNM stage.

Gastric lymph node metastasis

A Publication bias：

```
Begg's Test

  adj. Kendall's Score (P-Q) =       -7
          Std. Dev. of Score =     5.32
           Number of Studies =        6
                          z  =    -1.32
                  Pr > |z| =    0.188
                          z  =     1.13 (continuity corrected)
                  Pr > |z| =    0.260 (continuity corrected)
```

Egger's test

Std_Eff	Coef.	Std. Err.	t	P>|t|	[95% Conf. Interval]	
slope	3.267544	.7355231	4.44	0.011	1.225404	5.309684
bias	-3.5007	1.812534	-1.93	0.126	-8.5331	1.5317

B Sensitivity analysis：

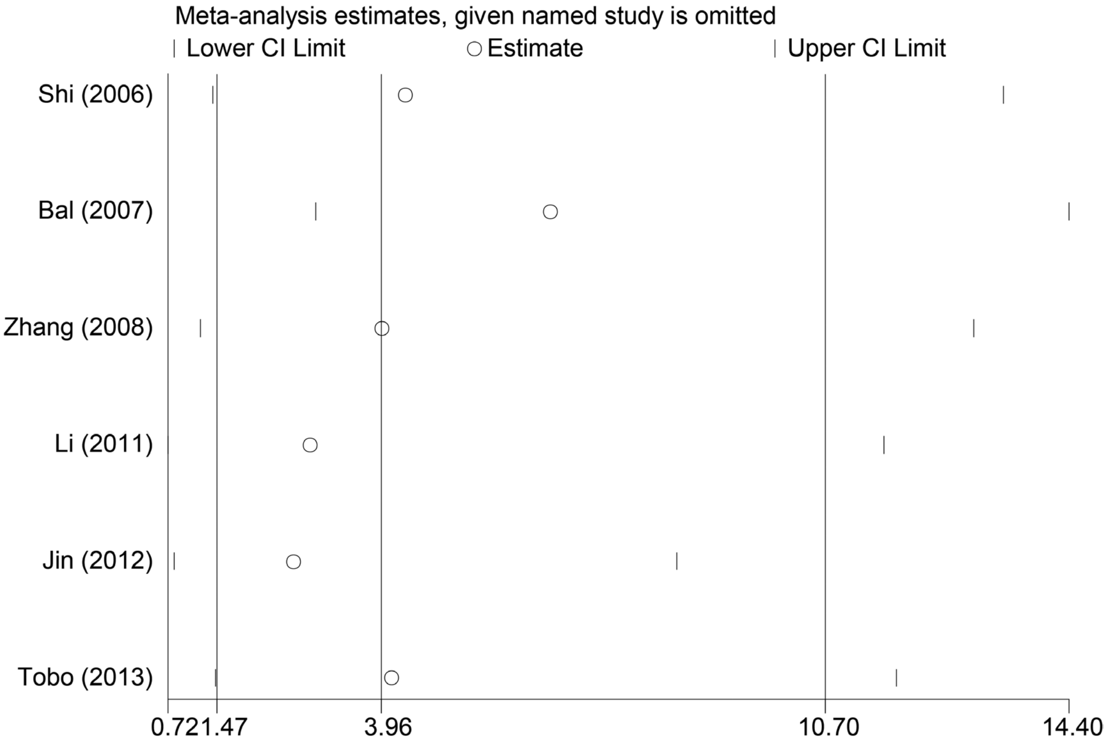

Supplementary Figure 5: (**A**) No evidence of significant publication bias was found in gastric lymph node metastasis. (**B**) Sensitivity analysis showed that no single study significantly influenced the pooled results of gastric lymph node metastasis.

Gastric distant metastasis

A Publication bias：

```
Begg's Test

  adj. Kendall's Score (P-Q) =       1
          Std. Dev. of Score =    1.91
           Number of Studies =       3
                          z  =    0.52
                    Pr > |z| =   0.602
                          z  =    0.00 (continuity corrected)
                    Pr > |z| =   1.000 (continuity corrected)
```

Egger's test

Std_Eff	Coef.	Std. Err.	t	P>|t|	[95% Conf.	Interval]
slope	-1.377463	5.028712	-0.27	0.830	-65.27331	62.51839
bias	4.567115	9.489831	0.48	0.714	-116.0126	125.1469

B Sensitivity analysis：

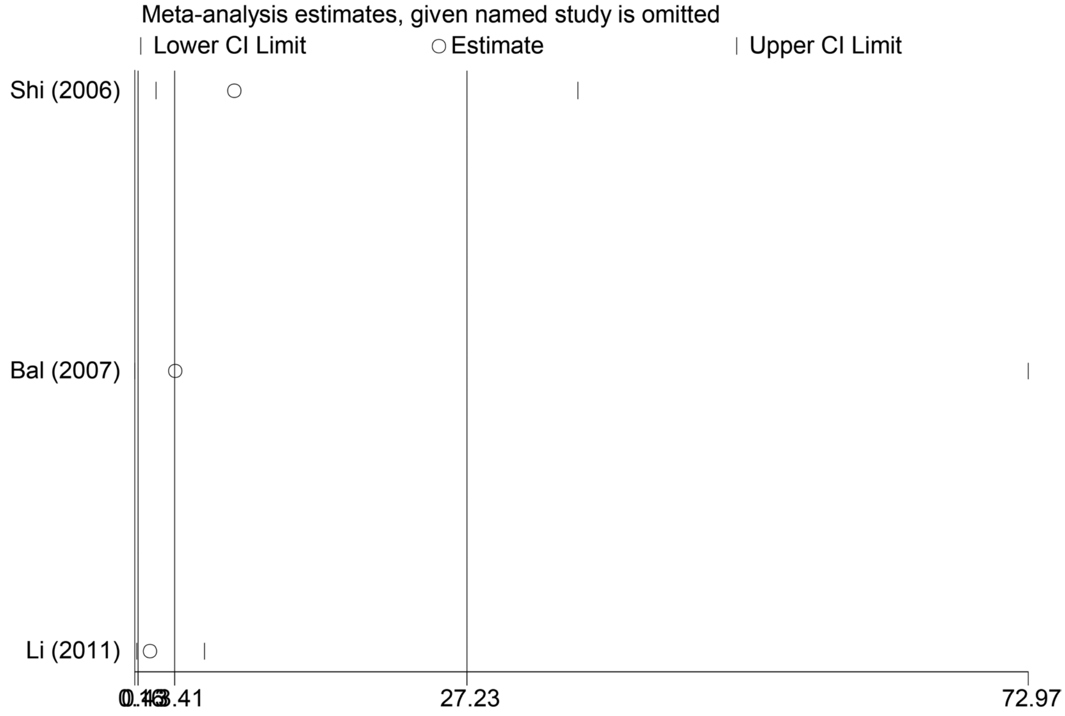

Supplementary Figure 6: (**A**) No evidence of significant publication bias was found in gastric distant metastasis. (**B**) Sensitivity analysis showed that no single study significantly influenced the pooled results of gastric distant metastasis.

Colorectal tumor grade

A Publication bias：

```
Begg's Test

  adj. Kendall's Score (P-Q) =      -2
          Std. Dev. of Score =    4.08
           Number of Studies =       5
                          z  =   -0.49
                   Pr > |z|  =   0.624
                          z  =    0.24 (continuity corrected)
                   Pr > |z|  =   0.806 (continuity corrected)
```

Egger's test

Std_Eff	Coef.	Std. Err.	t	P>|t|	[95% Conf.	Interval]
slope	1.664961	1.356572	1.23	0.307	-2.652257	5.982179
bias	-.3968041	1.884261	-0.21	0.847	-6.393363	5.599755

B Sensitivity analysis：

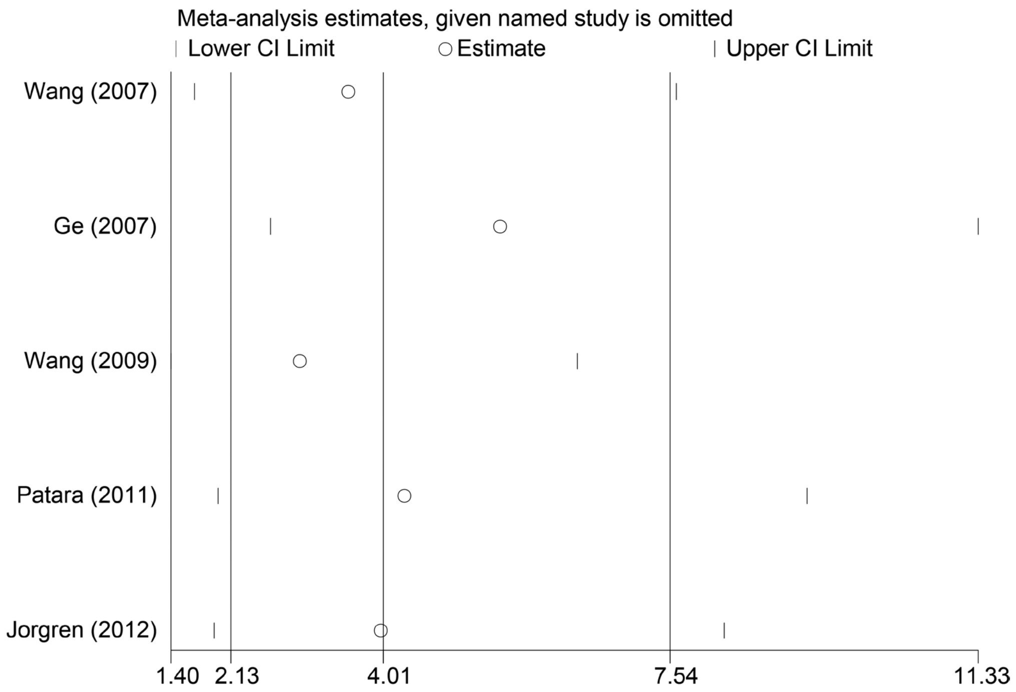

Supplementary Figure 7: (**A**) No evidence of significant publication bias was found in colorectal tumor grade. (**B**) Sensitivity analysis showed that no single study significantly influenced the pooled results of colorectal tumor grade.

Colorectal TNM stage

A Publication bias:

```
Begg's Test

  adj. Kendall's Score (P-Q) =       0
          Std. Dev. of Score =    2.94
           Number of Studies =       4
                          z  =    0.00
                    Pr > |z| =   1.000
                          z  =   -0.34 (continuity corrected)
                    Pr > |z| =   1.000 (continuity corrected)
```

Egger's test

Std_Eff	Coef.	Std. Err.	t	P>|t|	[95% Conf.	Interval]
slope	-2.197775	1.259084	-1.75	0.223	-7.615176	3.219626
bias	5.233321	1.807665	2.90	0.101	-2.544433	13.01107

B Sensitivity analysis:

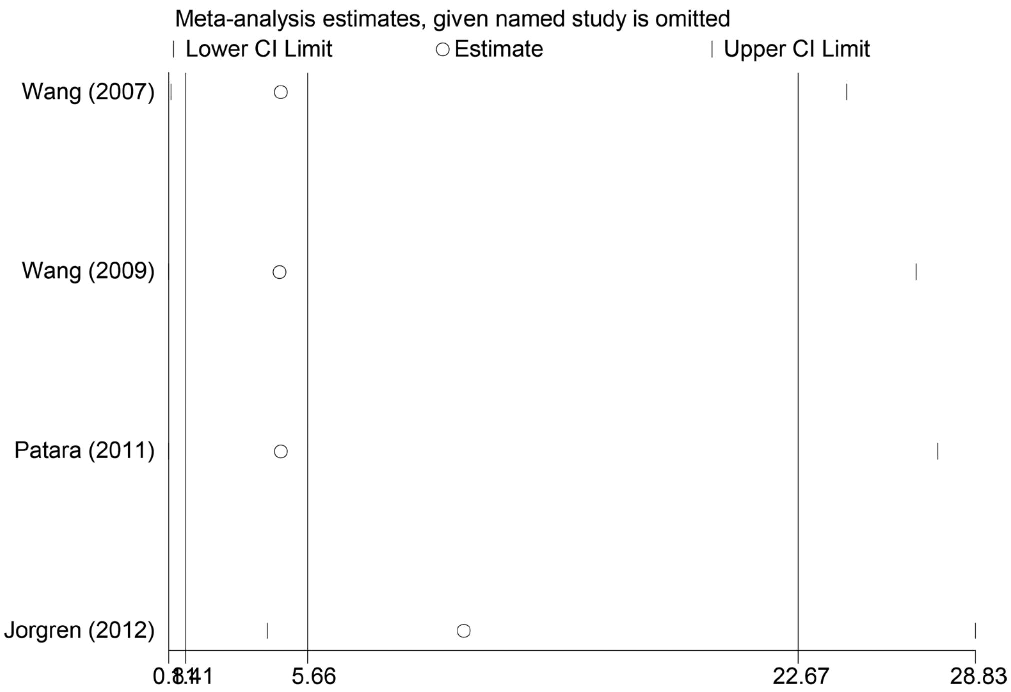

Supplementary Figure 8: (**A**) No evidence of significant publication bias was found in colorectal TNM stage. (**B**) Sensitivity analysis showed that no single study significantly influenced the pooled results of colorectal TNM stage.

Colorectal lymph node metastasis

A Publication bias：

```
Begg's Test

  adj. Kendall's Score (P-Q) =        3
          Std. Dev. of Score =     1.91
           Number of Studies =        3
                          z  =     1.57
                 Pr > |z|  =     0.117
                          z  =     1.04 (continuity corrected)
                 Pr > |z|  =     0.296 (continuity corrected)
```

```
Egger's test
```

Std_Eff	Coef.	Std. Err.	t	P>|t|	[95% Conf. Interval]	
slope	1.372831	.4188149	3.28	0.189	-3.948717	6.69438
bias	1.141133	.5354634	2.13	0.279	-5.662575	7.94484

B Sensitivity analysis：

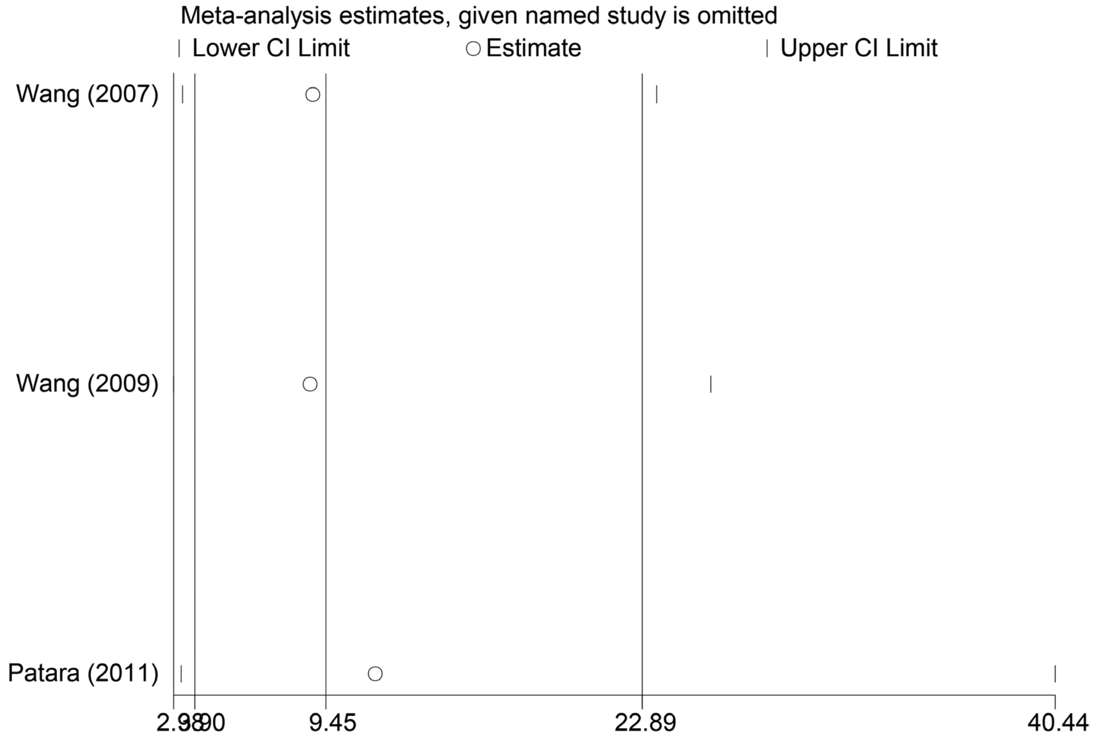

Supplementary Figure 9: (**A**) No evidence of significant publication bias was found in colorectal lymph node metastasis. (**B**) Sensitivity analysis showed that no single study significantly influenced the pooled results of colorectal lymph node metastasis.

Colorectal distant metastasis

A Publication bias:

```
Begg's Test

  adj. Kendall's Score (P-Q) =      -1
          Std. Dev. of Score =    1.91
           Number of Studies =       3
                          z  =   -0.52
                  Pr > |z|  =   0.602
                          z  =    0.00 (continuity corrected)
                  Pr > |z|  =   1.000 (continuity corrected)
```

Egger's test

Std_Eff	Coef.	Std. Err.	t	P>|t|	[95% Conf.	Interval]
slope	2.131333	4.409167	0.48	0.713	-53.89245	58.15511
bias	-2.038375	9.210979	-0.22	0.861	-119.075	114.9982

B Sensitivity analysis:

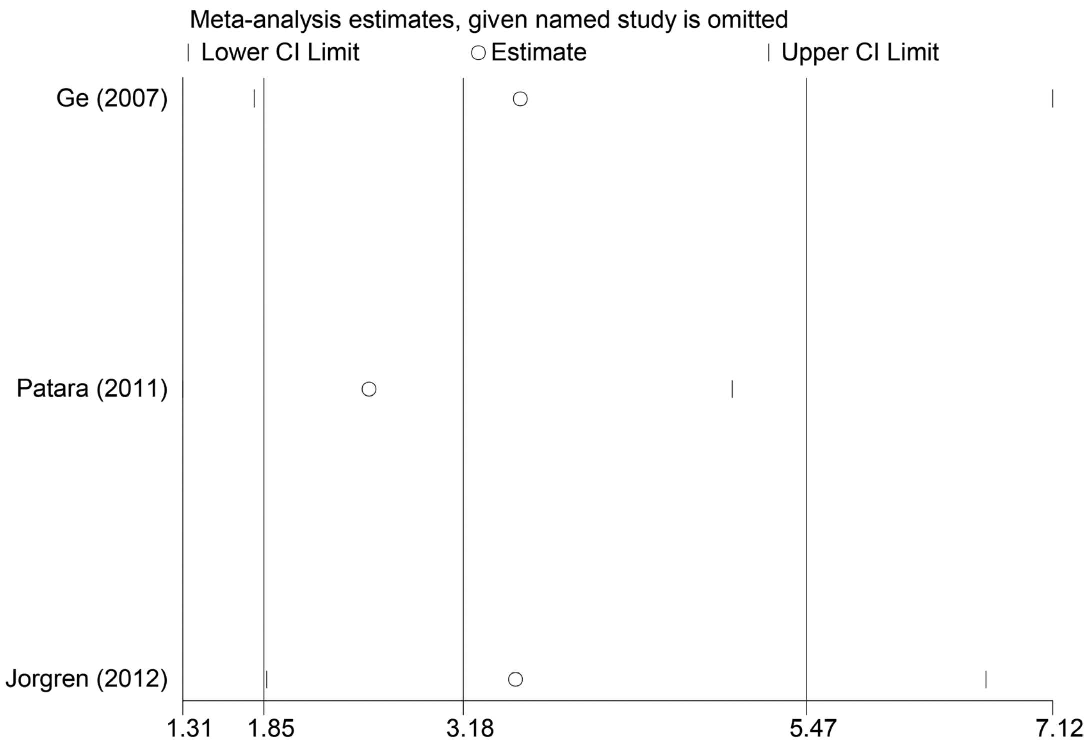

Supplementary Figure 10: (**A**) No evidence of significant publication bias was found in colorectal distant metastasis. (**B**) Sensitivity analysis showed that no single study significantly influenced the pooled results of colorectal distant metastasis.

Supplementary Table 1: Sensitivity analysis by omitting a single study

Cancer		Pooled OR (95% CI)	
		Before omitting	After omitting
**Gastric (omitting Zhang 2008 [19])**	Lymph node metastasis	3.96 (1.47, 10.70)	3.97 (1.21, 12.96)
**Colorectal (omitting Jorgren 2012 [29])**	Tumor grade	3.94 (2.10, 7.38)	3.94 (2.08, 7.48)
	TNM stage	5.66 (1.41, 22.67)	11.08 (4.26, 28.83)
	Distant metastasis	3.06 (1.77, 5.31)	3.40 (1.78, 6.50)

Note: Zhang 2008 [19] was only included in the meta-analysis of Erzin expression and lymph node metastasis of gastric cancer. Jorgren 2012 [29] was included in the meta-analysis of Erzin expression and tumor grade, TNM stage, distant metastasis of colorectal cancer.

Supplementary Table 2: PRISMA 2009 Checklist. See Supplementary_Table_2
